# Anthropometric cutoffs and associations with visceral adiposity and metabolic biomarkers after spinal cord injury

**DOI:** 10.1371/journal.pone.0203049

**Published:** 2018-08-31

**Authors:** Ryan M. Sumrell, Thomas E. Nightingale, Liron S. McCauley, Ashraf S. Gorgey

**Affiliations:** 1 Spinal Cord Injury Service and Disorders, Hunter Holmes McGuire VA Medical Center, Richmond, VA, United States of America; 2 Physical Medicine and Rehabilitation, Virginia Commonwealth University, Richmond, VA, United States of America; University of Northern British Columbia, CANADA

## Abstract

**Background/Objectives:**

To examine associations of different anthropometric measurements of central adiposity to visceral adipose tissue (measured via multi-axial magnetic resonance imaging; MRI) and cardiometabolic disease risk factors in men with spinal cord injury (SCI). Additionally, to determine population-specific seated/supine waist and abdominal circumference cutoffs, which may identify men at increased risk of cardiometabolic disease.

**Participants/Methods:**

Twenty-two men with chronic SCI underwent MRI scans, anthropometric measurements along with assessments of various cardiometabolic risk biomarkers. Pearson/part (accounting for age as a covariate) correlation coefficients were calculated to determine the associations between study variables. Abdominal and waist circumference cutoffs were extrapolated using the slope of linear regression equations.

**Results:**

Seated/supine abdominal and waist circumferences were (*P* < 0.01) associated with MRI visceral fat cross-sectional area (VAT_CSA_), VAT volume and CSA:Total_CSA_. Low density lipoprotein, non-high-density lipoprotein and total cholesterol were positively associated with seated/supine abdominal and waist circumferences after controlling for age; *r* = 0.50–0.61, *r* = 0.46–0.58, *r* = 0.52–0.58, *P* < 0.05, respectively. Tumor necrosis factor alpha was associated with seated/supine abdominal and waist circumferences after accounting for age; *r* = 0.49–0.51 and *r* = 0.48–0.56, *P* < 0.05 respectively. The population-specific cutoffs were 86.5cm and 88.3cm for supine waist and abdominal circumferences, respectively, as well as 89cm and 101cm for seated waist and abdominal circumferences, respectively. After dichotomizing VAT_CSA_ (< or ≥ 100cm^2^), peak oxygen uptake, triglycerides, insulin sensitivity and glycated hemoglobin were different (*P* < 0.05) between groups. After dichotomizing (< or ≥ 86.5cm) supine waist circumference, VAT_CSA_, triglycerides and insulin sensitivity were different *(P* < 0.05) between groups.

**Conclusions:**

Seated/supine circumferences are associated with both central adiposity and biomarkers of cardiometabolic disease risk in persons with SCI. Population-specific cutoffs are proposed herein to identify central adiposity and potential cardiometabolic disease risk after SCI.

## Introduction

Obesity is rapidly becoming a serious problem in persons that have physical disabilities, with a 1.2 to 3.9-fold increase in prevalence compared to the general population [1]. Specifically, in persons with spinal cord injury (SCI), the best data available would suggest that two in every three persons with SCI are likely to be obese [2]. The mechanisms by which obesity occurs in persons with SCI are multifactorial and these physiological processes have been eloquently described previously by the conceptual model of Disability-Associated Low Energy Expenditure Deconditioning Syndrome [3]. Following SCI, significant atrophy of paralyzed skeletal muscle and mobility impairments lead to a reduction in basal metabolic rate (BMR) and physical activity energy expenditure (PAEE), respectively [4,5]. Alterations in these key components of energy expenditure contributes to an energy surplus, which if maintained, favors the development of obesity. It has also been suggested that social factors (e.g. comfort food provided by family/friends), functional challenges (e.g. problems encountered when preparing food), physical barriers (e.g. transport to shops and supermarket store shelving) and environment (e.g. hospital food) also contribute to an ‘obesogenic environment’ in this population [6]. Obesity has been addressed as one of the key modifiable risk factors for the development of type II diabetes mellitus and cardiovascular disease (CVD) in persons with SCI [7] and, likely drives the increased prevalence of these chronic diseases compared to able-bodied individuals [8].

The accumulation of adipose tissue in different depots is altered in persons with SCI. In comparison to able-bodied controls, visceral adipose tissue (VAT) quantified by single slice computed tomography (CT) at the umbilical and L4-L5 levels have been shown to be 34% [9] and 45% (58% after adjusting for weight differences) [10] greater in persons with SCI. VAT_CSA_, measured by single-slice CT scan, has shown to be positively correlated with homeostasis model assessment of insulin resistance (HOMA-IR) and fasting insulin concentrations in persons with SCI [9, 10]. Gorgey et al, [11], demonstrated that VAT cross-sectional area (VAT_CSA_) quantified across multi-axial slices compared to a single-axial CSA is adequately describing the true associations between central adiposity and metabolic profile. These authors also demonstrated that VAT_CSA_ was positively and negatively associated with fasting plasma glucose (FPG) and insulin concentrations, respectively, but not post-load indices of insulin resistance measured during an oral glucose tolerance test. As FPG concentrations have been shown to correlate with basal rates of hepatic glucose output [12] and HOMA-IR predominantly reflects hepatic insulin sensitivity [13], the associations between VAT and peripheral insulin resistance in persons with SCI remains to be assessed using a more sensitive measure of skeletal muscle/adipose tissue insulin sensitivity (i.e. intravenous glucose tolerance test; IVGTT).

While previous research in this population has used gold-standard VAT imaging techniques (i.e. MRI and CT), barriers such as the high cost, accessibility to scanner and requirement for trained personnel often limit the sample size of such research studies and the feasibility of use in clinical settings. Although body mass index (BMI) is the most commonly used measure of obesity in the SCI literature [14], it is not an appropriate tool to use with this population given the deleterious body composition changes that occur post injury [4]. In the general population, waist and hip circumferences have been used as surrogate measures for central (i.e. VAT) and gluteofemoral (i.e. SAT) adiposity. The National Cholesterol Education Program report (NCEP) defined waist circumference cutoffs for obesity > 102cm, based on epidemiological evidence using able-bodied men [15]. However, these standard cutoffs may not be appropriate for persons with SCI. In Japanese men with SCI, supine waist circumference ≥ 81.3cm has been suggested to correspond to a VAT_CSA_ ≥ 100cm^2^ [16]. Moreover, in males and females with SCI, supine waist circumference ≥ 94cm has been proposed as the optimal cutoff for identifying increased CVD risk in this population specifically [17]. Despite waist circumferences being correlated to a range of cardiometabolic disease risk biomarkers [18] and its ease of use in multiple settings, there remains ambiguity as to which position (supine vs. seated) and cutoff best represents central adiposity and adverse CVD risk profiles in persons with SCI. Conflicting information regarding obesity in persons with SCI is due to, in part, the inconsistent use of obesity measurement tools or classification cutoffs that have not been validated for use in this population. Therefore, the purpose of this study is to identify the associations between: (i) seated/supine anthropometric measurements of central adiposity and VAT_CSA_ (quantified by the gold-standard multi-axial slice MRI); (ii) seated/supine anthropometric measurements of central adiposity and a range of biomarkers of cardiometabolic disease and, (iii) propose new population specific cutoffs for both seated/supine anthropometrics. This study aimed to identify the most adequate anthropometric measurement that is likely to detect both central obesity and elevated CVD risk in persons with SCI, while also proposing suitable population specific obesity cutoffs.

## Materials and methods

### Participants

Twenty-two men, aged between 18–50 years old, BMI ≤ 30kg/m^2^, with chronic motor complete SCI (C5-T11; American Spinal Cord Injury Classification A or B) participated in the TEREX-SCI trial (registered at clinicaltrials.gov: NCT01652040) [19]. Only cross-sectional baseline data is presented in this manuscript. The McGuire Veteran Affairs Investigation Research Board and the Virginia Commonwealth University (VCU) Office of Research and Innovation approved the current study. A neurological examination was performed per the International Standards for Neurological Classification of SCI (ISNCSCI) to determine the American Spinal Injury Association (ASIA) Impairment Scale (AIS) for each participant. Participants provided written, informed consent before the study commenced. Participants with the following pre-existing medical conditions were excluded: active urinary tract infection, ≥ stage 2 pressure ulcer, cardiovascular disease and/or uncontrolled type II diabetes mellitus.

### Anthropometric measurements

Participants were instructed to void their bladder before undergoing anthropometric measurements. Height was measured to the nearest 0.1cm by placing a wooden transfer board at the soles of the feet to ensure neutral dorsiflexion using a Harpenden Stadiometer while in a supine position. To establish each participants weight, the weight of the wheelchair was subtracted from the weight of the participant and wheelchair measured using a wheelchair weighing scale (Tanita, PW-63OU). Both waist and abdominal circumferences were measured in sitting and supine positions using a standard inflexible measuring tape (MFG, Lufkin, Executive Diameter Pocket Tape measure). Waist circumference was taken at the midpoint between the crest of the ilium and the inferior margin of the last rib. Abdominal circumference was measured at the level of the umbilicus. Supine hip circumference was also measured around the widest part of the trochanters. All measurements were made after the exhalation of a preceding deep breath. Values were recorded to the nearest 0.1cm and the mean of three values (within 0.5cm of each other) calculated. Supine waist circumference was divided by hip circumference to derive a waist-to-hip ratio for each participant. These anthropometric locations were chosen as they are commonly used in the wider SCI body composition literature [10, 20, 21, 22].

### Magnetic Resonance Imaging (MRI)

Abdominal MRI images were captured using a 1.5-Tesla magnet (General Electric, Waukesha, WI) whole body scanner, using a fast spin-echo sequence described previously [23]. Transverse images with a slice thickness of 0.8cm and inter-slice space of 1.2cm were captured from the xiphoid process to the femoral heads. Approximately 20–30 images were obtained depending on the length of the participant’s torso. Participants were instructed to remain as still as possible during the scan. To prevent any respiratory artefacts that could alter the quality of the images, participants were also asked to hold their breath for approximately 20 seconds. Images were sequenced anatomically using Image-J software (National Institute of Health, Bethesda, Maryland) and analyzed using specifically designed software (Win Vessel 2.0, Ronald Meyer, MSU). Each image was automatically segmented into fat and muscle, with bone and background tissue identified based on its signal intensity. Abdominal adipose tissue was separated into SAT and VAT (intraperitoneal and retroperitoneal fat) depots by an experienced technician, who manually highlighted regions of interest guided by anatomical landmarks. The cross-sectional areas (CSA’s) of these different compartments were used to derive VAT:SAT ratio. Total trunk CSA was defined as the total area within the outer border of the trunk. These data were used to normalize VAT_CSA_ to Total_CSA_ (VAT:Total ratio). All values were averaged across images to reflect the whole torso. VAT volume (cm^3^) was calculated by multiplying the CSA by the slice thickness (0.8cm) and inter-slice space (1.2cm).

### Indirect calorimetry and assessment of cardiometabolic disease risk biomarkers

Participants were woken up ~6.30 am, following a 12 hour overnight fast during an inpatient stay at a local hospital. Basal metabolic rate (BMR) was measured via indirect calorimetry using a portable metabolic system (COSMED K4b^2^, Rome, Italy) in a darkened, thermoneutral environment (ambient temperature between 20–25°C). The unit was calibrated prior to use per manufacturer’s instructions. Following calibration, a canopy was placed over the participant’s head as they lay in a supine position, with continuous breath-by-breath measurements made over a 20-minute period. Gas exchange values for the first five minutes were discarded, with BMR (kcal/day) averaged over the last 15 minutes. Following the measurement of BMR a cannula was inserted into an antecubital vein. Fasting whole-blood samples were drawn into serum separator and potassium oxalate/sodium fluoride tubes and centrifuged to collect serum and plasma samples, respectively. Lipid profiles (serum high-density lipoprotein cholesterol; HDL-C, Low-density lipoprotein cholesterol; LDL-C, Total cholesterol and triglycerides; TG) and plasma free-fatty acids (FFAs) were analyzed using enzymatic colorimetric quantification. Inflammatory biomarkers (Tumor necrosis factor α; TNF-α, Interleukin-6; IL-6 and c-reactive protein; CRP) were also analyzed in serum samples by enzyme-linked immunosorbent assays (ELISA) (ALPACO; Salem, NH).

Another cannula was inserted into the opposite arm to facilitate an intravenous glucose tolerance test (IVGTT), whereby participants were administered glucose (0.3g/kg intravenously over 30 seconds) followed by a bolus of insulin (0.02U/kg) twenty minutes later. Blood samples were drawn from the opposite arm every 1–3 minutes for the first 30 minutes, 5–10 minutes for the next 40 minutes and every 20 minutes for the next 80 minutes. The intravenous cannula was kept patent through periodic flushing with 0.9% NaCI. Plasma insulin and glucose were measured by ELISA (ALPACO; Salem, NH) and using a biochemistry analyzer, respectively. Glucose effectiveness (S_g_) and insulin sensitivity (S_i_) were determined using the MinMod software (MinMod Inc., Pasadena, CA) as described previously [24] (Diabetes Technology and Therapeutics). Glycated hemoglobin (HbA1c) was measured using a standard procedure in the VCU pathology laboratory. Peak lower extremity oxygen uptake (VO_2_ peak) was assessed in a subsample of participants (n = 15) at the point of fatigue on a FES bike (Restorative Therapies, RTI-300) during an incremental exercise protocol whereby the bike resistance was manually increased every 2 minutes (1, 3, 5, 7 Nm) until fatigue, defined by dropping the cycling rate to 18 revolutions per minute [25, 26]. Electrical stimulation electrodes were applied bilaterally to the quadriceps, hamstrings and gluteal muscles to facilitate cycling of the paralyzed lower extremities.

### Statistical analysis

All data were analyzed for normality of distribution. The distributions of TG, CRP, TNF-α, S_i_ and S_g_ were positively skewed. Thus, these data were log-transformed to permit the use of parametric statistics. IL-6 was negatively skewed and therefore reflected prior to log-transformation. Age, time since injury, level of injury, height, and weight were assessed as covariates through bivariate correlations with MRI outcomes, anthropometric measurements of central adiposity and biomarkers of cardiometabolic disease. Linear regression models were used to examine the relationship between the measures of central adiposity (MRI outcomes and anthropometric measurements) and biomarkers of cardiometabolic disease. From this, Pearson correlation coefficients (*r*) and part correlations, accounting for age, were chosen to assess the associations between MRI outcomes, anthropometric measurements of central adiposity and biomarkers of cardiometabolic disease. The following descriptors were used to help interpret the magnitude of each correlation: small (*r* > 0.1), moderate (*r* > 0.3), large (*r* > 0.5) and, very large (*r* > 0.7) [27, 28]. Where significant correlations were observed for VAT_CSA_ with biomarkers of cardiometabolic disease, participants were dichotomized into two groups using accepted cut-points for abdominal obesity; < 100cm^2^ (n = 11) or ≥ 100cm^2^ (n = 11) [11]. Seated and supine abdominal and waist circumference cutoffs, equivalent to 100cm^2^ VAT_CSA,_ were calculated using linear regression equations to derive population-specific waist circumference cutoffs. Participants were also dichotomized based on supine and seated anthropometrics to examine the effects on VAT and various biomarkers of cardiometabolic disease using the newly developed cutoffs. Two-tailed independent T-Tests were used to determine significant differences between groups for parameters of central adiposity and various biomarkers of cardiometabolic disease. Statistical analyses were performed using SPSS (SPSS Statistics version 24, IBM Corp, Armonk, USA). Statistical significance was accepted at a priori of α ≤ 0.05.

## Results

Participant demographics, injury characteristics, anthropometric measurements, MRI outcomes and biomarkers of cardiometabolic disease are presented in Table 1. Fifty-nine percent of participants had depressed HDL-C (< 35mg/dL), whereas only 9% of participants had elevated levels of total cholesterol (≥ 200mg/dL) and LDL-C (> 130mg/dL). Central adiposity was apparent in 48%, 27% and 5% of participants using the following cut-points; **≥** 100cm^2^ MRI VAT_CSA_, supine waist circumference **≥** 94cm (SCI-specific) and **≥** 102cm (NCEP), respectively. Moreover, fifty-nine percent of participants were at high risk of developing future coronary events (CRP > 3000ng/mL).

**Table 1:** Participant demographics, injury characteristics, MRI outcomes, anthropometric measurements and biomarkers of cardiometabolic disease.

	Total (n = 22)	Paraplegic (n = 14)	Tetraplegic (n = 8)
** *Demographics***			
**Age (y)**	36 ± 10	35 ± 9	37 ± 12
** *Injury Characteristics***			
**Lesion Level**^a^	C5 –T11	T4 –T11	C5 –C7
**Time Since Injury**^a^ **(y)**	8 ± 8	8 ± 9	8 ± 7
** *Anthropometric Measurements***			
**Body Mass (kg)**	78.4 ± 13.4	80.3 ± 13.0	75.2 ± 14.3
**BMI**	24.9 ± 4.0	25.3 ± 3.5	24.1 ± 5.0
**Seated Abdominal Circumference (cm)**	100.3 ± 13.5	100.0 ± 14.9	100.8 ± 11.6
**Seated Waist Circumference (cm)**	88.8 ± 9.3	89.8 ± 10.9	87.0 ± 5.8
**Supine Abdominal Circumference (cm)**	87.6 ± 12.6	86.4 ± 11.8	89.8 ± 14.3
**Supine Waist Circumference (cm)**	85.9 ± 11.8	85.0 ± 11.6	87.5 ± 12.8
**Supine Hip Circumference (cm)**	97.5 ± 10.0	97.1 ± 10.0	98.2 ± 10.6
**Waist:Hip Ratio**	0.88 ± 0.09	0.88 ± 0.10	0.89 ± 0.08
** *MRI Outcomes***			
**VAT**_**CSA**_ **(cm**^**2**^**)**^a^	100.4 ± 61.0	89.8 ± 63.0	117.5 ± 57.2
**VAT Volume (cm**^**3**^**)**^a^	5287 ± 3409	4550 ± 3327	6485 ± 3401
**VAT**_**CSA**_**/TOTAL**^a^	0.15 ± 0.08	0.10 ± 0.10	0.20 ± 0.10
**VAT:SAT Ratio**^a^	0.73 ± 0.44	0.69 ± 0.43	0.84 ± 0.44
** *Blood Pressure***			
**SYS (mm/Hg)**	117.2 ± 19.5	123.4 ± 18.0	106.4 ± 18.1
**DIA (mm/Hg)**	72.8 ± 9.9	75.2 ± 8.5	68.6 ± 11.4
** *Whole-body Outcomes***			
**VO**_**2**_ **peak (L/min)**^c^	0.54 ± 0.20	0.61 ± 0.20	0.41 ± 0.12
**BMR (Kcal/day)**	1137 ± 280	1216 ± 278	1022 ± 240
** *Lipid Profile***			
**LDL-C (mg/dL)**	92.6 ± 26.9	89.3 ± 29.1	98.4 ± 23.2
**HDL-C (mg/dL)**	35.0 ± 8.1	36.1 ± 8.9	33.0 ± 6.5
**Non-HDL (mg/dL)**	115.1 ± 27.7	108.1 ± 30.8	127.4 ± 16.7
**Total Cholesterol (mg/dL)**	150.1 ± 29.0	144.3 ± 31.9	160.4 ± 21.1
**TG (mg/dL)**	111.1 ± 54.5	93.9 ± 37.6	141.1 ± 68.2
**FFA (pg/mL)**	363.7 ± 188.8	334.5 ± 178.6	394.1 ± 216.6
** *Carbohydrate Profile***			
**HbA1c (%)**^a^	5.40 ± 0.50	5.20 ± 0.40	5.60 ± 0.50
**S**_**i**_ **[(mu/L)^l.min^-1]**^b^	8.6 ± 6.3	10.5 ± 5.7	4.6 ± 6.1
**S**_**g**_ **[min^-1]**	0.02 ± 0.01	0.02 ± 0.01	0.02 ± 0.00
** *Inflammatory Markers***			
**CRP (ng/mL)**	7221 ± 6574	7650 ± 6572	7393 ± 6953
**TNF-α (pg/mL)**	15.3 ± 8.0	15.5 ± 8.2	16 ± 8.2
**IL-6 (pg/mL)**	5.9 ± 6.8	6.9 ± 7.7	5.0 ± 5.4

Basal metabolic rate; BMR, body mass index; BMI, c-reactive protein; CPR, cross-sectional area; CSA, diastolic blood pressure; DIA, free fatty acid; FFA, glucose effectiveness; S_g_, glycated hemoglobin; HbA1c, high-density lipoprotein cholesterol; HDL-C, insulin sensitivity; S_i_, interleukin-6; IL-6, low-density lipoprotein cholesterol; LDL-C, magnetic resonance imaging; MRI, peak oxygen uptake; VO_2_ peak, subcutaneous adipose tissue; SAT, systolic blood pressure; SYS, triglycerides; TG, tumor necrosis factor alpha; TNF-α, visceral adipose tissue cross-sectional area; VAT_CSA_. Missing data:

^a^ n = 21

^b^ n = 19

^c^ n = 15.

### Age as a covariate

Age was significantly (*P* < 0.01) associated with anthropometric (seated and supine waist and abdominal circumferences) (*r* = 0.66–0.70) and MRI indices (*r* = 0.69–0.82) of central adiposity. Age was also positively associated with TG (*r* = 0.52, *P* < 0.05) and HbA1c (*r* = 0.47, *P* < 0.05), and negatively associated with VO_2_ peak (*r* = -0.66, *P* < 0.01) and S_i_ (*r* = -0.48, *P* < 0.05).

### Magnetic-resonance imaging outcomes and biomarkers of cardiometabolic disease

Pearson correlations and part correlations (accounting for age) between MRI outcomes and biomarkers of cardiometabolic disease are shown in Table 2. All MRI outcomes of central adiposity are positively associated with HbA1c (*r* = 0.47–0.69, *P* < 0.05) and TG (*r* = 0.50–0.59, *P* < 0.05). Significant negative associations (*P* < 0.05) were observed between all MRI outcomes; S_i_ and VO_2_ peak (except for VAT:SAT ratio). HDL-C was negatively correlated with CSA/total and VAT:SAT ratio, the later association remaining significant when accounting for age (*r* = -0.39, *P* < 0.05). After accounting for age, there were significant associations (*P* < 0.05) between TNF-α; VAT_CSA_ and VAT volume of *r* = 0.46 and *r* = 0.48, respectively.

**Table 2:** Linear regression models were used to find Pearson correlation coefficients and part correlations, with adjustments for age as a covariate, between magnetic-resonance imaging outcomes of central adiposity and biomarkers of cardiometabolic disease.

	Pearson correlation coefficients (*r*)				Part correlation coefficients (*r*), accounting for age			
	VAT_CSA_ (cm^2^)	VAT volume (cm^3^)	CSA/Total	VAT:SAT	VAT_CSA_ (cm^2^)	VAT volume (cm^3^)	CSA/Total	VAT:SAT
** *Blood Pressure***								
**SYS**	0.01	0.08	-0.10	-0.15	-0.18	-0.05	-0.35	-0.25
**DIA**	0.32	0.33	0.34	0.37	0.08	0.11	0.11	0.14
** *Whole body physiology***								
**VO**_**2**_ **peak**	**-0.62†**	**-0.60†**	**-0.64†**	-0.51	-0.07	-0.09	-0.08	0.00
**BMR**	-0.37	-0.30	**-0.46†**	-0.44	-0.22	-0.10	-0.37	-0.31
** *Lipid profile***								
**LDL-C**	0.05	0.02	-0.05	-0.28	0.19	0.13	0.03	-0.22
**HDL-C**	-0.39	-0.38	**-0.49†**	-0.60*	-0.21	-0.20	-0.40	**-0.39†**
**Non-HDL**	0.29	0.28	0.21	0.03	0.28	0.24	0.13	-0.09
**Total cholesterol**	0.17	0.16	0.06	-0.14	0.21	0.18	0.01	-0.19
**TG**^**+**^	**0.51†**	**0.50†**	0.59*	0.57*	0.17	0.15	0.30	0.25
**FFA**	0.33	0.35	0.31	0.22	0.45	0.44	0.41	0.16
** *Carbohydrate Profile***								
**HbA1c**	**0.49†**	**0.47†**	**0.56†**	0.69*	0.19	0.16	0.31	**0.40†**
**S**_**i**_^**+**^	**-0.53†**	**-0.55†**	-0.66*	**-0.56†**	-0.27	-0.31	**-0.51†**	-0.24
**S**_**g**_^**+**^	-0.08	-0.07	-0.23	-0.42	0.17	0.13	-0.10	-0.27
** *Inflammatory markers***								
**CRP**^**+**^	0.20	0.23	0.28	-0.01	0.11	0.17	0.24	-0.13
**TNF-α**^**+**^	0.30	0.33	0.30	0.13	**0.46†**	**0.48†**	0.44	0.10
**IL-6**^**-**^	0.04	0.10	0.09	0.07	0.16	0.25	0.23	0.12

Basal metabolic rate; BMR, c-reactive protein; CRP, cross-sectional area; CSA, diastolic blood pressure; DIA, free fatty acid; FFA, glucose effectiveness; S_g_, glycated hemoglobin; HbA1c, high-density lipoprotein cholesterol; HDL-C, insulin sensitivity; S_i_, interleukin-6; IL-6, low-density lipoprotein cholesterol; LDL-C, peak oxygen uptake; VO_2_ peak, triglycerides; TG, tumor necrosis factor alpha; TNF-α, subcutaneous adipose tissue; SAT, systolic blood pressure; SYS, Visceral adipose tissue cross-sectional area; VAT_CSA_.

^+^ positive skew (data was log transformed prior to analysis)

^-^ negative skew (data was reflected and log transformed prior to analysis).

†P < 0.05

*P < 0.01.

### Magnetic-resonance imaging outcomes and anthropometric measurements of trunk adiposity

**Fig 1:**
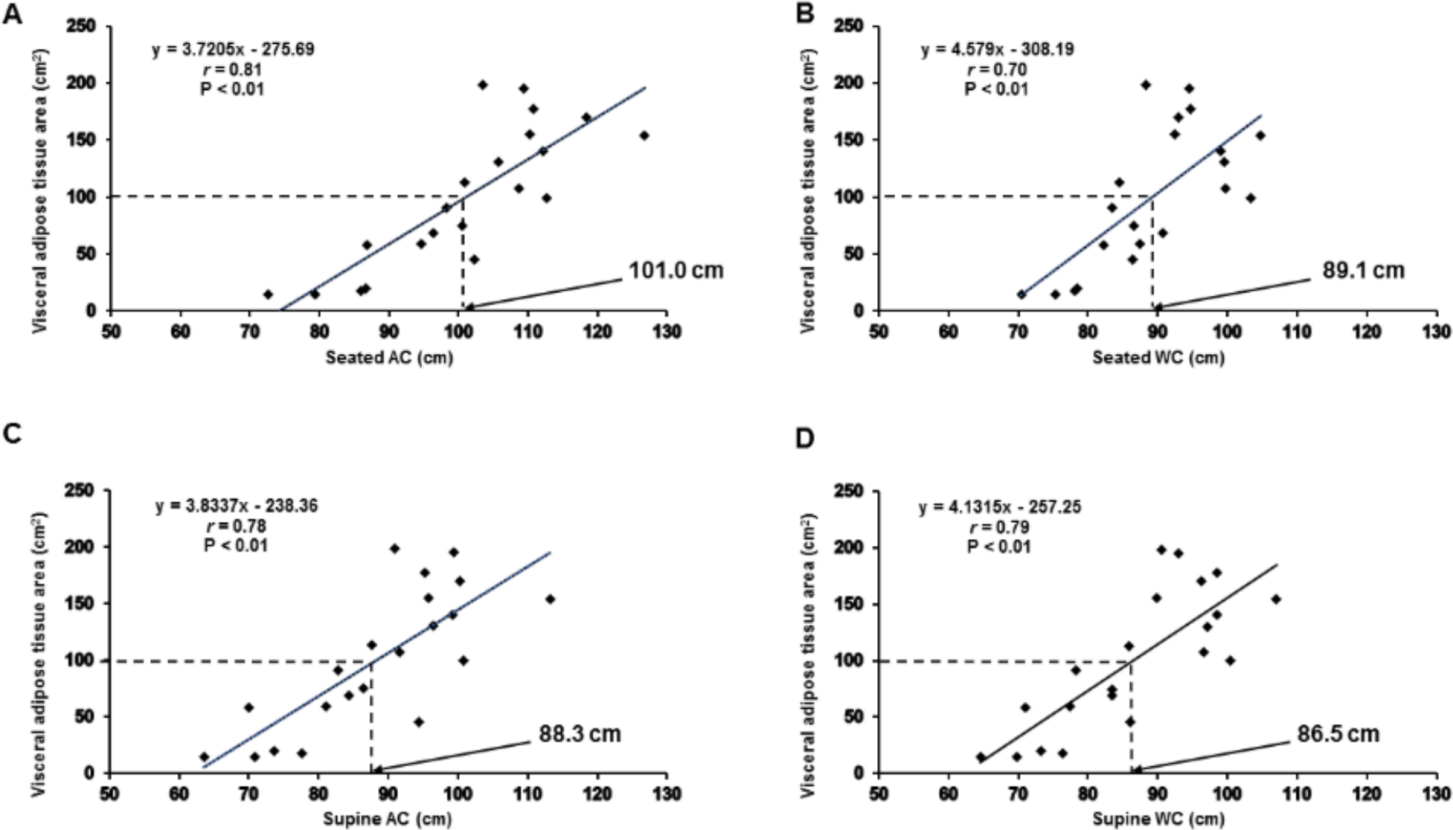
Associations between magnetic-resonance imaging (MRI) visceral adipose tissue cross-sectional area (VAT_CSA_) and anthropometric measurements of central adiposity; seated abdominal (A) and waist (B) circumference and, supine abdominal (C) and waist (D) circumference. Lines of best fit (fixed) are shown for each correlation, along with Pearson correlation co-efficient values (*r*), significance (*P*) and specific linear regression equations. Population specific cutoffs, corresponding to 100cm^2^ VAT_CSA_, are highlighted by the dashed lines.

Seated and supine abdominal circumferences showed similar *(P* < 0.01) associations with VAT_CSA_ (*r* = 0.81 and 0.78, respectively) and VAT volume (*r* = 0.82 and 0.79, respectively). Compared to seated waist circumference, supine waist circumferences were more strongly associated with VAT_CSA_ (*r* = 0.79 vs. *r* = 0.70) and VAT volume (*r* = 0.80 vs. *r* = 0.68). Supine hip circumference was not significantly (*P* > 0.05) associated to measurements of VAT. The only anthropometric index significantly associated with VAT:SAT ratio was waist:hip ratio (*r* = 0.74, *P* < 0.01). The seated waist circumference cutoffs for a VAT_CSA_ of 100cm^2^, estimated from the developed regression equations, are presented in Fig 1.

### Anthropometric measurements of trunk adiposity and biomarkers of cardiometabolic disease

Supine anthropometric measurements of central adiposity demonstrated consistent, moderate and large associations with lipid profile components (Table 3). Most of these associations remained significant even when accounting for age. Seated abdominal circumference and supine abdominal and waist circumferences revealed moderate negative correlations with S_i_ (range; *r* = -0.45 –-0.49, *P < 0*.*05*), while seated waist circumference had small correlations (*r* = -0.29, *P* > 0.05). There were also significant part correlations, accounting for age, between TNF-α and anthropometric measurements of central adiposity (range; *r* = 0.48–0.56, *P* < 0.05).

**Table 3:** Linear regression models were used to find Pearson correlation coefficients and part correlations, with adjustments for age as a covariate, between anthropometric measurements of central adiposity and biomarkers of cardiometabolic disease.

	Pearson correlation coefficients (*r*)						Part correlation coefficients, accounting for age					
	Seated		Supine				Seated		Supine			
	AC (cm)	WC (cm)	AC (cm)	WC (cm)	HC (cm)	Waist:hip ratio	AC (cm)	WC (cm)	AC (cm)	WC (cm)	HC (cm)	Waist:hip ratio
** *Blood Pressure***												
**SYS**	0.05	0.19	0.11	0.19	0.27	-0.01	0.04	0.24	0.13	0.24	0.27	-0.04
**DIA**	0.10	0.23	0.09	0.25	-0.07	**0.44†**	-0.12	0.08	-0.11	0.09	-0.15	0.37
** *Whole-body Physiology***												
**VO**_**2**_ **peak**	-0.05	0.15	-0.40	-0.44	-0.22	-0.39	-0.05	0.15	0.06	0.06	-0.05	0.16
**BMR**	-0.08	0.06	-0.05	-0.07	0.32	-0.23	0.17	0.33	0.18	0.38	**0.42†**	-0.04
** *Lipid Profile***												
**LDL-C**	0.35	0.42	0.33	0.33	**0.48†**	-0.03	0.55*	0.61*	**0.50†**	**0.54†**	**0.51†**	0.02
**HDL-C**	-0.16	-0.11	-0.06	-0.08	0.21	-0.34	0.07	0.12	0.19	0.20	0.31	-0.18
**Non-HDL**	0.55*	**0.53†**	**0.50†**	**0.51†**	**0.49†**	0.20	0.58*	**0.54†**	**0.50†**	**0.54†**	**0.46†**	0.10
**Total cholesterol**	**0.48†**	**0.47†**	**0.46†**	**0.47†**	**0.53†**	0.10	0.58*	**0.55†**	**0.53†**	0.57*	**0.52†**	0.04
**TG**^**+**^	**0.51†**	0.34	**0.44†**	**0.45†**	0.09	**0.53†**	0.19	-0.09	0.11	0.10	-0.07	0.22
**FFA**	0.10	0.09	0.17	0.20	-0.10	0.36	0.09	0.08	0.15	0.19	-0.14	0.44
** *Carbohydrate Profile***												
**HbA1c**	0.29	0.16	0.20	0.27	-0.16	**0.52†**	-0.07	-0.22	-0.16	-0.11	0.31	0.24
**S**_**i**_^**+**^	**-0.49†**	-0.29	**-0.47†**	**-0.45†**	-0.16	-0.44	-0.18	0.13	-0.14	-0.07	0.01	-0.08
**S**_**g**_^**+**^	0.11	0.09	0.14	0.07	0.35	-0.24	0.31	0.27	0.33	0.26	0.41	-0.20
** *Inflammatory markers***												
**CRP**^**+**^	0.18	0.09	0.10	0.10	0.02	0.13	0.13	0.01	0.17	0.21	0.05	0.21
**TNF-α**^**+**^	**0.44†**	0.43	0.36	0.38	0.25	0.28	**0.51†**	**0.48†**	**0.49†**	0.56*	0.27	0.37
**IL-6**^**-**^	-0.02	-0.05	-0.05	-0.09	-0.03	-0.08	0.04	-0.01	0.09	0.06	0.03	0.05

Abdominal circumference; AC, basal metabolic rate; BMR, c-reactive protein; CRP, diastolic blood pressure; DIA, free fatty acid; FFA, glucose effectiveness; S_g_, glycated hemoglobin; HbA1c, high-density lipoprotein cholesterol; HDL-C, hip circumference; HC, insulin sensitivity; S_i_, interleukin 6; IL-6, low density lipoprotein cholesterol; LDL-C, peak oxygen uptake; VO_2_ peak, systolic blood pressure; SYS, triglycerides; TG, tumor necrosis factor alpha; TNF-α, waist circumference; WC.

^+^ positive skew (data was log transformed prior to analysis)

^-^ negative skew (data was reflected and log transformed prior to analysis).

†P < 0.05

* P < 0.01.

### Differences in biomarkers of cardiometabolic disease using central obesity cutoffs

**Fig 2:**
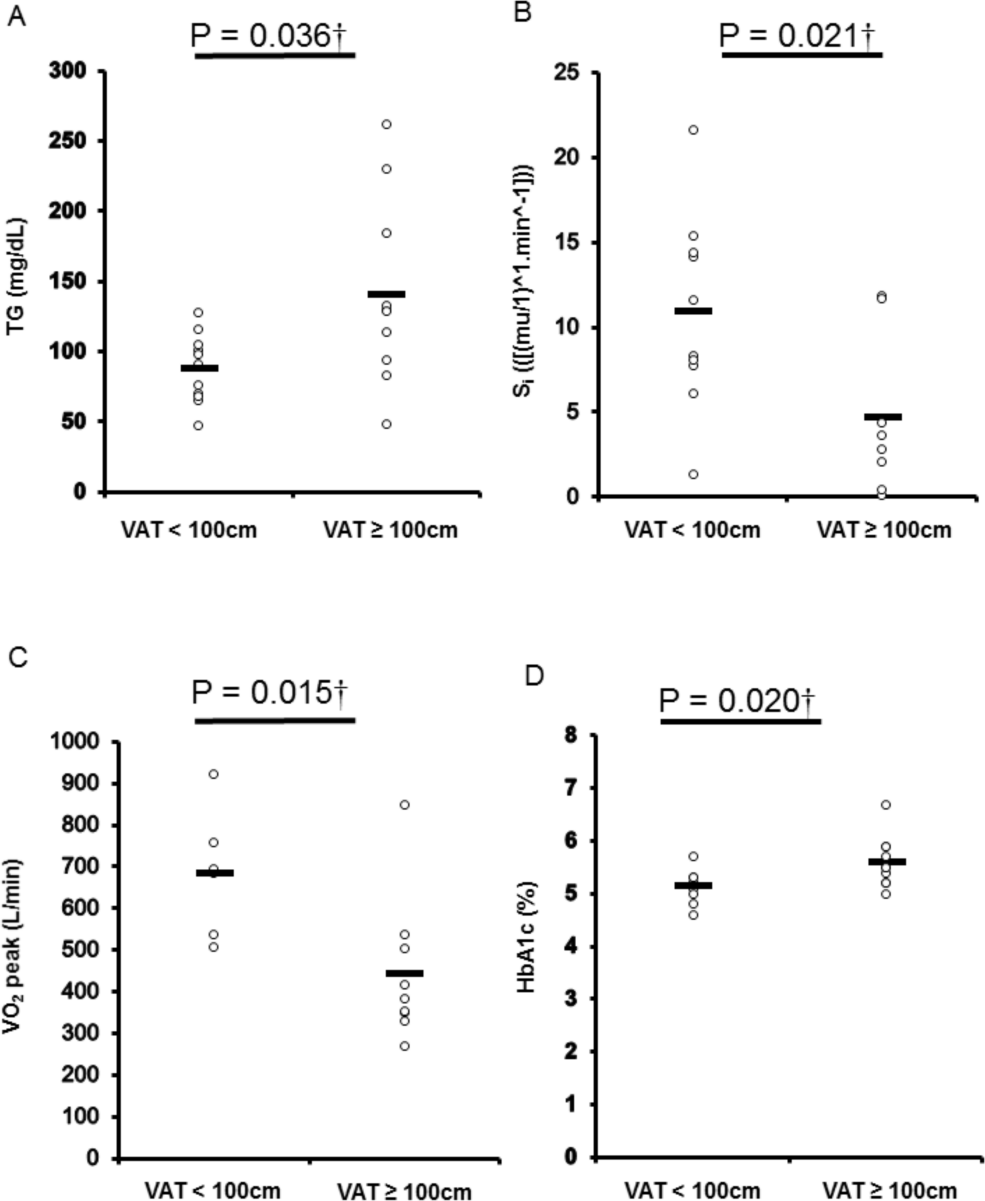
Differences in cardiometabolic disease risk biomarkers [triglycerides (TG) (A), insulin sensitivity (S_i_) (B), glycated hemoglobin (HbA1c) (C) and peak oxygen uptake (VO_2_ peak) (D) among participants dichotomized per multi-axial magnetic resonance imaging (MRI) visceral adipose tissue cross-sectional area (VAT_CSA_) (< 100cm^2^, n = 11; ≥ 100cm^2^, n = 10). *P* values are shown for significant differences (*P* < 0.05).

**Fig 3:**
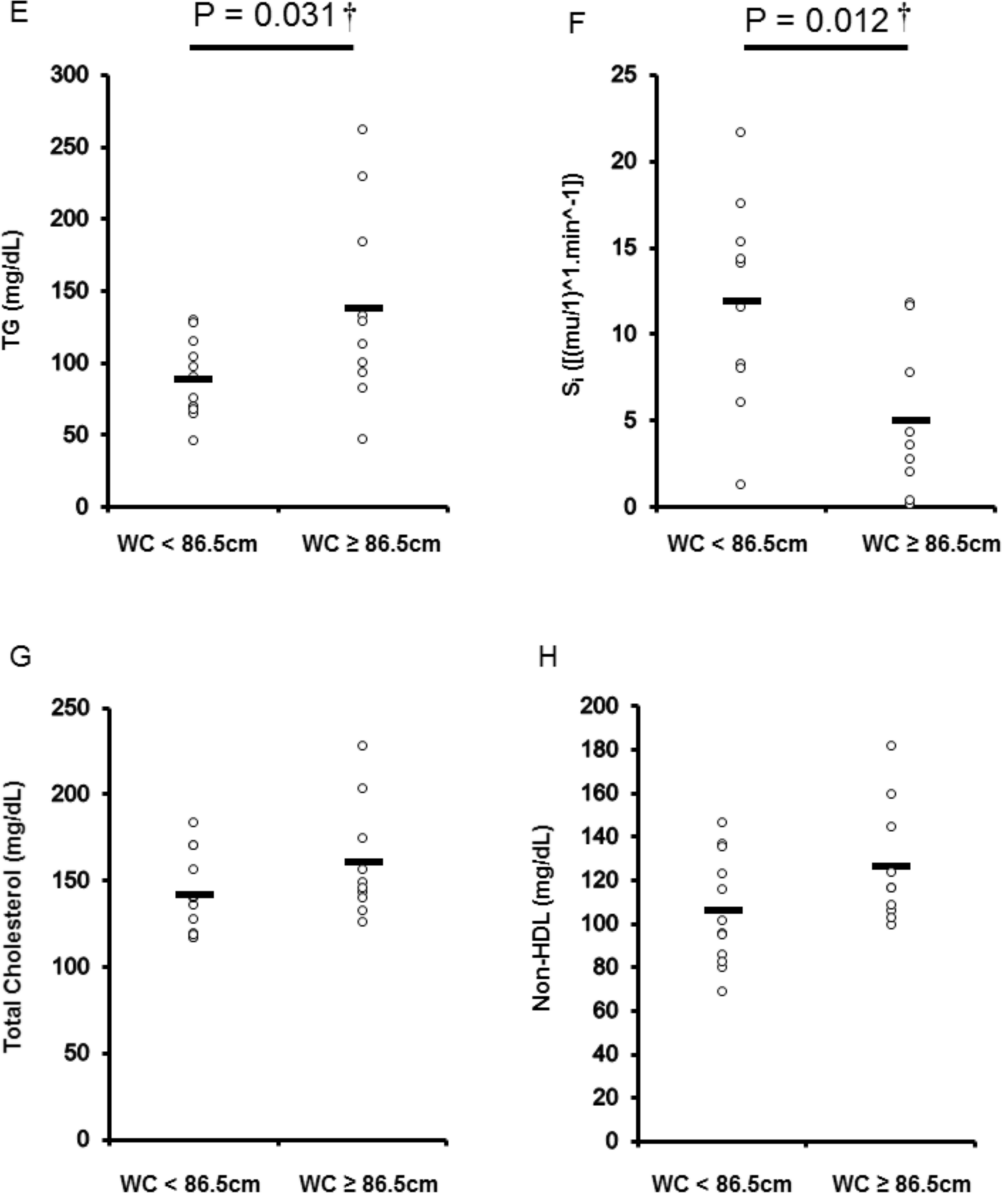
Differences in cardiometabolic disease risk biomarkers [triglycerides (TG) (E), insulin sensitivity (S_i_) (F), total cholesterol (G), Non-HDL (H) among participants dichotomized per the derived supine waist circumference cutoff for central adiposity (< 86.5cm, n = 12; ≥ 86.5cm, n = 10). *P* values are shown for significant differences (*P* < 0.05).

VAT_CSA_ and supine waist circumferences were significantly associated with specific biomarkers of cardiometabolic disease (Table 3). Participants were dichotomized into two groups using either established (Fig 2) or population-specific cutoffs (Fig 3). Participants with VAT_CSA_ cutoff ≥ 100cm^2^ had significantly greater TG (*P* = 0.036) and HbA1c (*P* = 0.020), and reduced S_i_ values (*P* = 0.021) (Fig 2). Participants with VAT_CSA_ ≥ 100cm^2^ also had lower VO_2_ peak than those with central adiposity < 100cm^2^. Using our specific supine waist circumference cutoff point of 86.5cm, TG and S_i_ were significantly (*P* < 0.05) different between both dichotomized groups (Fig 3). Table 4 also compares central adiposity and cardiometabolic disease risk factors between groups dichotomized using cutoffs generated for other anthropometric measurements.

**Table 4:** Generated population specific seated/supine AC and WC cutoff points with averaged and standard deviation for the cardiometabolic risk factors chosen in the current study.

	Seated				Supine			
*Anthropometric Measurements*	AC < 101 (cm)	AC ≥ 101 (cm)	WC < 89.1 (cm)	WC ≥ 89.1 (cm)	AC < 88.3 (cm)	AC ≥ 88.3 (cm)	WC < 86.5 (cm)	WC ≥ 86.5 (cm)
**Body Mass (kg)**	72.3 ± 11.7	**84.6 ± 12.6†**	72.3 ± 11.2	8**5.9 ± 12.5**†	72.3 ± 11.7	**84.6 ± 12.6†**	72.6 ± 11.2	**85.4 ± 13†**
**BMI (cm)**	22.2 ± 3.6	27.5 ± 2.4*	22.6 ± 3.6	27.6 ± 2.7*	22.2 ± 3.6	27.5 ± 2.4*	22.43± 3.5	27.8 ± 2.3*
**Seated AC (cm)**	89.7 ± 9.2	111.0 ± 6.9*	91.3 ± 10.1	111.1 ± 7.9*	89.7 ± 9.2	111.0 ± 6.9*	90.7 ± 9.5	118.8 ± 6.6*
**Seated WC (cm)**	81.6 ± 5.8	96.0 ± 5.9*	81.8 ± 5.4	97.2 ± 4.8*	81.6 ± 5.8	96.0 ± 5.9*	82.0 ± 5.7	97.0 ± 5.2*
**Supine AC (cm)**	77.3 ± 7.8	97.9 ± 6.1*	79.3 ± 9.5	97.6 ± 7.4*	77.3 ± 7.8	97.9 ± 6.1*	78.7 ± 8.9	98.3 ± 6.3*
**Supine WC (cm)**	76.0 ± 6.6	95.8 ± 5.8*	77.4 ± 7.8	96.1 ± 6.3*	76.0 ± 6.6	95.8 ± 5.8*	76.8 ± 6.9	96.8 ± 5.0*
**Supine HC (cm)**	92.3 ± 6.7	**102.7 ± 10.1†**	92.7 ± 6.7	**103.2 ± 10.5†**	92.3 ± 6.7	**102.7 ± 10.1†**	92.8 ± 6.7	**103.2 ± 10.6†**
**Waist:Hip ratio**	0.8 ± 0.1	0.9 ± 0.1*	0.8 ± 0.1	0.9 ± 0.1*	0.8 ± 0.1	0.9 ± 0.1*	0.8 ± 0.1	0.9 ± 0.1*
***MRI Outcomes***								
**MRI VAT**_**csa**_ **(cm**^**2**^**)** ^a^	53.3 ± 35.2	143.2 ± 45.6*	64.4 ± 55.4	139.9 ± 39.0*	53.3 ± 35.2	143.2 ± 45.6*	52.6 ± 33.4	152.9 ± 33.9*
**MRI VAT Volume (cm**^**3**^**)** ^a^	3154 ± 2252	7578 ± 2665*	3413 ± 3051	7932 ± 1966*	2767 ± 2111	7873 ± 2604*	2690 ± 2018	8143 ± 1997*
**MRI VAT**_**csa**_**/Total** ^a^	0.09 ± 0.06	0.19 ± 0.07*	0.11 ± 0.08	**0.19 ± 0.05†**	0.09 ± 0.06	0.19 ± 0.07*	0.09 ± 0.05	0.20 ± 0.06*
**MRI VAT:SAT ratio** ^a^	0.52 ± 0.27	**0.86 ± 0.44†**	0.58 ± 0.38	0.83 ± 0.40	0.52 ± 0.27	**0.86 ± 0.44†**	0.50 ± 0.26	**0.92 ± 0.42†**
***Blood Pressure***								
**SYS (mm/Hg)**	116 ± 21	119 ± 19	117 ± 19	117 ± 21	116 ± 21	119 ± 19	116 ± 20	119 ± 20
**DIA (mm/Hg)**	71 ± 10	74 ± 11	72 ± 10	74 ± 10	81 ± 14	74 ± 11	71 ± 9	76 ± 10
***Whole-body Outcomes***								
**VO**_**2**_ **peak (L/min)** ^c^	0.65 ± 0.23	0.48 ± 0.17	0.59 ± 0.23	0.51 ± 0.19	0.65 ± 0.23	0.48 ± 0.17	0.65 ± 0.20	0.46 ± 0.17
**BMR (Kcal/day)**	1058 ± 227	1215 ± 316	1051 ± 218	1240 ± 321	1058 ± 227	1215 ± 316	1053 ± 217	1238 ± 324
***Lipid Profile***								
**LDL-C (mg/dL)**	85.1 ± 19.3	100.1 ± 32.0	86.6 ± 22.3	99.8 ± 31.2	85.1 ± 19.3	100.1 ± 32.0	88.08 ± 21.1	98 ± 32.9
**HDL-C (mg/dL)**	35.8 ± 9.2	34.2 ± 7.2	35.3 ± 9.2	34.7 ± 7.0	35.8 ± 9.2	34.2 ± 7.2	35.9 ± 8.8	33.9 ± 7.5
**Non-HDL (mg/dL)**	102.1 ± 23.0	**128.2 ± 26.7†**	105.7 ± 25.5	126.5 ± 27.1	102.1 ± 23.0	**128.2 ± 26.7†**	105.8 ± 25.5	126.3 ± 27.3
**Total Cholesterol (mg/dL)**	137.9 ± 20.6	**162.4 ± 31.9†**	140.9 ± 24.1	161.2 ± 31.7	137.9 ± 20.6	**162.4 ± 31.9†**	141.8 ± 23.7	160.2 ± 32.8
**TG (mg/dL)**	85.1 ± 25.0	**137.0 ± 64.2†**	95.3 ± 38.6	129.9 ± 66.2	85.1 ± 25.0	**137.0 ± 64.2†**	88.8 ± 26.9	137.9 ± 67.6
**FFA (pg/mL)**	354.2 ± 193.5	373.2 ± 192.8	353.8 ± 201.3	375.6 ± 182.5	354.2 ± 193.5	373.2 ± 192.8	335.25 ± 195.8	397.8 ± 184.1
***Carbohydrate Profile***								
**HbA1c (%)** ^a^	5.21 ± 0.26	5.47 ± 0.57	5.2 ± 0.24	5.54 ± 0.61	5.21 ± 0.26	5.47 ± 0.57	5.2 ± 0.25	5.51 ± 0.58
**S**_**i**_ **[(mu/L)^l.min^-1]** ^b^	11.6 ± 6.3	**5.9 ± 5.1†**	11.6 ± 6.3	**5.3 ± 4.4†**	11.6 ± 6.3	**5.9 ± 5.1†**	11.9 ± 6.0	**5.0 ± 4.5†**
**S**_**g**_ **[min^-1]**	0.02 ± 0.01	0.02 ± 0.01	0.02 ± 0.01	0.02 ± 0.01	0.02 ± 0.01	0.02 ± 0.01	0.02 ± 0.01	0.02 ± 0.01
***Inflammatory Markers***								
**CRP (ng/mL)**	5774 ± 6841	8668 ± 6273	6420 ± 6846	8182 ± 6457	5774 ± 6841	8668 ± 6273	5455 ± 6615	9340 ± 6182
**TNF-α (pg/mL)**	11.7 ± 5.6	**19.0 ± 8.5†**	12.7 ± 6.8	18.5 ± 8.4	11.7 ± 5.6	**19.0 ± 8.5†**	11.3 ± 5.4	**20.1 ± 8.1†**
**IL-6 (pg/mL)**	4.9 ± 4.9	6.9 ± 8.4	6.0 ± 5.8	5.8 ± 8.1	4.9 ± 4.9	6.9 ± 8.4	4.6 ± 4.8	7.5 ± 8.6

Abdominal circumference; AC, basal metabolic rate; BMR, body mass index; BMI, c-reactive protein; CPR, cross-sectional area; CSA, diastolic blood pressure; DIA, free fatty acid; FFA, glucose effectiveness; S_g_, glycated hemoglobin; HbA1c, high-density lipoprotein; HDL-C, hip circumference; HC, insulin sensitivity; S_i_, interleukin-6; IL-6, low-density lipoprotein; LDL-C, magnetic resonance imaging; MRI, peak oxygen uptake; VO_2_ peak, subcutaneous adipose tissue; SAT, systolic blood pressure; SYS, triglycerides; TG, tumor necrosis factor alpha; TNF-α, visceral adipose tissue cross sectional area; VAT_CSA_, waist circumference; WC. Missing data:

^a^ n = 21

^b^ n = 19

^c^ n = 15.

†P < 0.05

*P < 0.01.

## Discussion

The current study was undertaken to propose various anthropometric cutoffs capable of distinguishing those who are at risk of developing central obesity and cardiometabolic disorders after SCI. Central obesity has previously been defined in men as a waist circumference measurement greater than 102cm [11]. Both seated or supine waist and abdominal circumferences were strongly associated with VAT and VAT:SAT ratio as measured by the gold standard MRI. However, it is important to identify population specific cutoffs for either seated or supine anthropometrics to identify individuals with elevated VAT and increased cardiometabolic disease risk. These cutoffs were 86.5cm and 88.3cm for supine waist and abdominal, respectively, as well as 89.1cm and 101cm for seated waist and abdominal, respectively. Both seated and supine anthropometrics are tightly associated with lipid profile and low grade inflammation (i.e. TNF-α), independent of age in persons with SCI. Parallel associations were established between VAT and cardiometabolic disease risk (HDL-C, HBA1c and TNF-α). Finally, the established supine waist circumference cutoff clearly distinguished between those who are at an increased risk of central adiposity, insulin resistance and dyslipidemia.

Cardiometabolic disorders are considered serious sequalae after of SCI. Approximately, 50–75% present with impaired glucose tolerance and are at high risk of developing type II diabetes mellitus [7]. Furthermore, 75% are at high risk of developing dyslipidemia, with 50% at risk of developing metabolic syndrome [29, 30]. These comorbidities are likely to impact quality of life and impose socioeconomic burden after SCI. Central adiposity characterized by increasing waist circumference, VAT_CSA_ or VAT:SAT ratio has been shown to be tightly associated with cardiometabolic disorders after SCI. Maki et al. and Nightingale et al. also found that waist circumference was correlated with TG and negatively correlated with serum HDL-C [18, 31] in persons with SCI. Controversies have been raised regarding the use of waist circumference as a surrogate for VAT_CSA_ in persons with SCI [10, 23]. However, in the current study, anthropometric measurements were tightly associated with VAT indices either during supine or seated positioning. VAT is tightly associated with indices of insulin resistance as quantified by single slice CT scan [9, 10] and multi-axial MRI [23]. Moreover, VAT has previously been negatively associated with HDL-C in persons with SCI [11]. Anecdotal evidence supports the hypothesis that VAT_CSA_ > 100cm^2^ is likely to promote a more pro-inflammatory state [32] due to the infiltration of immune cells (macrophages and T lymphocytes), which secrete inflammatory cytokines such as TNF-α and IL-6 [33, 34]. Therefore, the first prophylactic line of defense is establishing cutoffs that will flag those who are risk of developing cardiometabolic disorders after SCI.

Persons with SCI are likely to consume high fat diet; a predisposing lifestyle factor for dyslipidemia, central adiposity and insulin resistance [5, 23, 35, 36]. Groah et al, (2009) found that persons with paraplegia and tetraplegia consume 81.4g and 82.7g of fat per day, respectively, while the recommended daily intake for able-bodied individuals is 40-70g/d [35]. Elevated VAT was associated with impaired S_i_, calculated following the injection of a glucose bolus directly into the circulation. IVGTT measurements likely offer improved measurement of peripheral insulin resistance (i.e. adipose tissue and skeletal muscle). To our knowledge, this is the first study to demonstrate associations with indices of VAT and a more sensitive measure of peripheral insulin resistance after SCI. Insulin resistance leads to a decreased suppression of lipolysis in adipocytes, resulting in elevated concentrations of FFA in the systemic circulation. This creates a vicious cycle which further exacerbates insulin resistance in the liver and skeletal muscle [37]. In support of this, the current data demonstrated a strong negative association between FFA and S_g_ (*r* = -0.57, *P* = 0.007). Thus, the mechanisms behind VATs contribution to elevated insulin resistance may be related to its higher rate of lipolytic activity (i.e. increased flux of free fatty acids (FFA)) and/or its anatomical location (i.e. FFA output directly to the liver via the hepatic portal vein) [38]. In support of this notion, liver adiposity was found to be tightly associated with VAT, TG and non-HDL-C in persons with SCI. The increase in liver adiposity was also positively associated with fasting plasma glucose concentrations and HbA1c, and negatively associated with S_i_ [39].

With respect to lipid profile, the atherogenic effects observed in persons with SCI are well established [7,11,40]. The amplified risk of coronary heart disease in this population is likely a function of reduced HDL-C [41]. VAT promotes a more pro-inflammatory state [32] due to the infiltration of immune cells (macrophages and T lymphocytes) that secrete inflammatory cytokines such as TNF-α and IL-6 [33, 34]. The data herein, shows indices of central adiposity are positively associated with TNF-α after accounting for age. It is possible that the etiology of depressed HDL-C in persons with SCI is a result of the direct relationship between endothelial lipase (EL) and inflammatory cytokines (including TNF-α) [42, 43]. EL plays a role in the clearance of HDL-C from the circulation, by catalyzing the hydrolysis of HDL-C phospholipids [41], thereby linking chronic low-grade inflammation and lipid abnormalities in persons with SCI. Gorgey et al. also demonstrated a very large (*r* = 0.75) correlation between VAT and TG. This current study supports this association, with a large (*r* = 0.51) correlation between VAT_CSA_ and TG, and a similar correlation (*r* = 0.45) between supine waist circumference and TG. Additionally, low plasma HDL-C concentrations could also be associated with physical inactivity, which is commonly linked to chronic systemic inflammation and increased VAT accumulation [41]. Considering the nearly perfect (*r* = 0.82) association between age and VAT_CSA_, persons aging with SCI and central adiposity are at increased risk of metabolic abnormalities and should therefore be the focus of future research efforts in this population.

Establishing SCI specific anthropometric cutoffs may likely detect, earlier, those who have heightened risks of developing cardiometabolic disorders. This may also allow clinicians to provide early feedback about lifestyle behaviors (dietary habits and exercise). Anthropometric measurements have previously been used as a surrogate measure to estimate VAT accumulation in persons with SCI. Edwards et al. found that supine waist circumference at three locations (lowest rib, iliac crest and the midpoint between the lowest rib and iliac crest) was associated to VAT_CSA_ (*r* > 0.90) in persons with SCI [10]. Significant correlations were noted in the current study between anthropometric measurements of central adiposity and VAT_CSA_, which permitted the development of the seated/supine abdominal and waist circumference cutoffs, extrapolated from 100cm^2^ MRI VAT_CSA_. Previous research proposed that persons with SCI have 42% more VAT per cm of waist circumference than their able-bodied counterparts [10]. Therefore, using able-bodied cutoff points would misclassify many of the SCI population as low risk for cardiometabolic risk factors.

## Limitations

This study is limited by its cross-sectional design and small sample size. Thus, it is not possible to infer causality. However, the plethora of associations assessed, along with the use of both gold-standard imaging techniques and anthropometric measurements of central adiposity provides useful data to inform the assessment of obesity in persons with SCI. Although this study lacks generalizability to females with SCI, significant differences in VAT accumulation has been shown between the sexes (males 1.8–2.6 times greater VAT_CSA_ than females) [44], which may explain why men display elevated CVD risk profiles [45]. Consequently, to ensure a more homogenous population only males were recruited for this current study. Such associations remain to be assessed specifically in females with SCI. It is not possible to comment on the role of physical activity in increased cardiometabolic disease risk in this population, as this variable was not measured in the current study. However, a recent study demonstrated that body composition variables, and not level of physical activity, may explain poor metabolic profile after SCI [18]. Previous cross-sectional research advocated accounting for age to better understand the associations between central adiposity and biomarkers of cardiometabolic disease in persons with SCI [11]. Considering that VAT has been shown to be increased in middle-aged men in comparison to younger adults [46], also accounting for age as a covariate is a notable strength of this study.

Level of injury also plays a role in the accumulation of VAT as seen in Table 1. While paraplegic and tetraplegic participants had similar waist circumference measurements, VAT measurements differed between the groups. Participants with paraplegia on average had VAT_CSA_ 89.8cm^2^ while those with tetraplegia had on average VAT_CSA_ 117.5cm^2^. Future studies will therefore need to develop new waist circumference cutoffs to account for the differences between these two groups. Additionally, this is a small male cohort, with a mixture of 41% veterans and 59% non-veterans. Future studies are required to ascertain the validity of the newly developed anthropometric prediction equations across a larger cohort and females with SCI.

## Conclusion

Considering the deleterious impact of central adiposity on cardiometabolic health, as eluded to herein, it is imperative that researchers develop strategies to reduce VAT in persons with SCI. In the able-bodied population, the combination of regular physical activity and dietary manipulation (to generate a sustainable energy deficit) is the cornerstone of obesity and type II diabetes mellitus management [47, 48]. Considering how prevalent such conditions are in persons with SCI, we advocate a renewed impetus to expand upon the limited existing evidence-base concerning the combination of diet and exercise, to reduce VAT and promote concomitant improvements in cardiometabolic health in this population. The proposed cutoffs herein may better identify persons at risk of chronic cardiometabolic diseases than those used previously, specific to certain ethnicities. Determining whether men with SCI exceed these proposed site specific anthropometric cutoffs might help facilitate earlier identification of persons at increased risk of developing cardiometabolic diseases. Future studies are necessary to cross-validate our proposed anthropometric cutoffs of central adiposity in persons with SCI and ascertain their validity in a larger sample size as well as in a female cohort. It is hoped progress in this area allows practitioners to more easily and better identify persons at increased risk of chronic diseases and provide early prevention guidance.
